# Emotional Skills and Nursing Training: A Study on Italian Students and a TRI-COM-Based Educational Model

**DOI:** 10.3390/brainsci15090961

**Published:** 2025-09-03

**Authors:** Giulia Savarese, Giovanna Stornaiuolo, Alessandro Vertullo, Carolina Amato, Luna Carpinelli

**Affiliations:** 1 Department of Medicine, Surgery and Dentistry, University of Salerno, 84080 Baronissi, Italy; giovannastornaiuolo81@gmail.com (G.S.); alessandrovertullo@gmail.com (A.V.); lcarpinelli@unisa.it (L.C.); 2 Master’s Degree Course in Organizational Psychology, Marketing, and Human Resources, Università Cattolica del Sacro Cuore, 20123 Milan, Italy; carolina.amato@icatt.it

**Keywords:** emotional competence, nursing education, alexithymia, empathy, TRI-COM model

## Abstract

Background/Objectives: Emotional competence is a crucial skill in nursing education, yet it remains underdeveloped in academic curricula. This study aims to (1) propose and preliminarily apply the TRI-COM model as a conceptual and educational framework to operationalize the definition of emotions within nursing contexts, and (2) explore the multidimensional structure of emotional competence among nursing students. Methods: A cross-sectional study was conducted with 233 nursing students (M_age = 23.79, SD = 5.19) from various Italian universities, with 82.8% identifying as female. The participants completed an online questionnaire including the Toronto Alexithymia Scale (TAS-20) and the Jefferson Scale of Empathy—Health Profession Student (JSE-HPS) version. Descriptive statistics, t-test, ANOVA, and Pearson’s correlation analyses were performed. Results: The overall mean TAS-20 score was 60.36 (SD = 11.22), which is close to the clinical threshold (cut-off = 61). The EOT subscale showed the highest mean (M = 26.48, SD = 3.16), suggesting a tendency toward externally oriented thinking. The mean JSE-HPS total score was 87.05 (SD = 7.88), with higher scores for Perspective Taking (M = 60.95, SD = 7.42) than Compassionate Care (M = 18.92, SD = 6.40). A significant gender difference was found in Perspective Taking (female: M = 61.54, male: M = 58.10; *p* = 0.007). The ANOVA results showed no significant differences in empathy across academic years, but the DIF subscale of TAS-20 showed a trend near significance (*p* = 0.053). Significant negative correlations were observed between age and TAS-20 scores (e.g., age–TAS-20 total: r = −0.23, *p* < 0.001). Conclusions: The findings suggest a general tendency toward rationalization and emotional detachment among students, possibly as a coping strategy in emotionally demanding contexts. The TRI-COM model—an original pedagogical framework inspired by tripartite theories of emotion—was used as a conceptual lens, providing a preliminary framework to interpret emotional competence in nursing education. Further research is needed to validate its educational relevance and explore practical applications within curricula.

## 1. Introduction

Within the field of cognitive and affective sciences, numerous authors have emphasized the importance of distinguishing the immediate physiological experience of emotion from its subsequent narrative elaboration [1,2,3]. Emotions can arise without full conscious awareness, implicitly influencing decisions and behaviors. Only later, through integration with autobiographical memory and linguistic processes, do feelings emerge, understood as the subjective and reflective experience of emotion [4].

This distinction is crucial not only for psychological theory but also for the training of healthcare professionals, who must be able to recognize their own emotions, integrate them into the helping relationship, and regulate themselves adaptively when faced with critical events [5]. Unacknowledged emotions may trigger automatic responses, whereas conscious feelings enable intentional and relational actions.

The neuroscientific exploration of emotions remains hindered by the lack of a universally accepted definition. Competing theoretical models emphasize neurobiological, cognitive, affective, and social dimensions, complicating efforts to consolidate findings into a coherent framework. This ambiguity extends to practical contexts, particularly nursing, where emotions play an omnipresent yet rarely conceptualized role. Nurses constantly work in emotionally charged environments, sharing patients’ pain, fear, and hope. The ability to manage emotions, engage empathetically, and maintain personal well-being is increasingly critical for ensuring high-quality care. However, emotional competencies in nursing education are often developed informally through clinical exposure, without structured pedagogy or standardized assessment [6].

In this scenario, repeated exposure to situations perceived as uncontrollable or unmodifiable—such as persistent patient suffering, lack of resources, or the inability to meaningfully influence clinical outcomes—may foster the development of learned helplessness [7]. This psychological condition can induce increasing emotional passivity among nurses, reducing confidence in their own ability to act, and favoring the emergence of alexithymic traits as a defensive strategy. In this context, alexithymia may not only represent an individual vulnerability but also a maladaptive—although functionally protective—response to a chronically stressful and uncontrollable clinical environment.

To address this complexity, the present study adopts the TRI-COM model (Triple Component Model), which defines emotion as a multisystemic process consisting of three components: (1) physiological activation, (2) cognitive representation, and (3) motivational regulation [8]. The TRI-COM label and its educational application are original contributions of this study. However, the model draws theoretical inspiration from the tripartite conceptualization of emotional processing described in the neuroscientific literature. In particular, Singer et al. [8] proposed that empathy and emotion involve interacting components of physiological arousal, cognitive appraisal, and motivational-affective regulation. While they did not explicitly name this structure, their findings provide the conceptual foundation for the TRI-COM framework presented here.

The TRI-COM model is particularly useful in nursing education, as it supports a comprehensive understanding of emotions and their regulation, which is essential for training competent and emotionally resilient professionals. The first component, physiological activation, refers to bodily responses to emotional stimuli, such as increased heart rate, sweating, or muscle tension. Nursing students can learn to recognize these signals through psychoeducational modules focused on bodily awareness during stress. Such awareness is fundamental for monitoring emotional intensity in high-pressure situations, such as medical emergencies or surgical interventions. As shown by Sabine-Farrell et al. [9], psychoeducational training aimed at bodily awareness has demonstrated positive effects on stress management among healthcare professionals.

The second component of the TRI-COM model, cognitive representation, concerns the ability to label and understand one’s emotional experiences. In nursing, this involves metacognitive activities that encourage students to reflect on and name the emotions experienced in complex professional situations, such as contact with terminally ill patients. Effective strategies include the use of emotional diaries, where students record their daily emotional responses, as suggested by Kabat-Zinn [10], in mindfulness-based practices, which help healthcare professionals recognize and understand emotions without judgment. For example, a student may face difficulties in emotionally engaging with a patient who has received a severe diagnosis. Through reflective dialogue with a mentor or journaling, the student may learn to identify feelings of sadness or anxiety, improving awareness and emotional regulation in care settings.

The third component of the TRI-COM model, motivational regulation, refers to the capacity to manage emotions constructively through coping strategies such as deep breathing, positive visualization, or other self-care techniques. Nursing students can be trained to use these tools to maintain emotional balance in stressful situations, such as caring for critically ill patients or during prolonged shifts. As argued by Lazarus and Folkman [11], effective coping strategies significantly reduce stress and improve professional performance. A practical example in nursing education may include teaching relaxation techniques during high-fidelity simulations of surgical interventions, enabling students to maintain emotional control and reduce anxiety, thereby improving clinical performance.

In nursing, emotional competencies (ECs) are increasingly recognized as essential for professional effectiveness and patient care. Following Goleman’s perspective [12], literature often refers to emotional intelligence as a key component of nursing practice, particularly in fostering empathy, adaptability, and relational skills [13,14]. Nurses who are able to understand and regulate both their own and others’ emotions are more capable of reflecting on complex clinical situations, which in turn enhances problem-solving, critical thinking, and communication abilities [15,16]. Such reflection contributes not only to improved care quality and patient satisfaction but also to stress reduction and professional well-being [17,18].

However, while most of the existing literature links emotional intelligence to nursing competencies, our approach introduces an innovative perspective. We propose to move beyond Goleman’s framework [12] by applying the classical model of emotional competencies, conceptualized as a multidimensional construct with three components: physiological, cognitive, and motivational [19]. In this study, the TRI-COM model is proposed as a potential framework for structuring emotional competencies in nursing education. In this framework, an emotionally competent nurse is not only someone capable of managing emotions in practice, but also one who integrates physiological awareness, cognitive appraisal, and motivational drive into relational and professional contexts.

Unlike previous uses of neurobiological models in the context of basic research, the TRI-COM framework is intended as a pedagogical guide. Its originality lies in the translation of classical emotion theory into a concrete educational strategy tailored to the realities of nursing training. By framing emotional competencies within the TRI-COM model, we intend to establish a new theoretical foundation for ECs in nursing, bridging classical theories of emotions with contemporary challenges in healthcare education. This innovation represents our original contribution, distinguishing our work from the prevailing EI-focused literature, and offering a concrete educational pathway for developing ECs in nursing curricula.

Furthermore, the TRI-COM model provides a valuable theoretical framework for understanding phenomena such as alexithymia, which describes the disconnection between emotional experience and awareness. Nurses with high levels of alexithymia may struggle to recognize their own emotional states, potentially compromising care effectiveness in emotionally intense contexts such as end-of-life care or chronic illness management [20]. In this sense, the TRI-COM model offers a pathway to bridge this gap, training students to identify and express their emotions in healthy ways. Conversely, empathy—as the integration of cognitive and motivational dimensions of emotional awareness—emerges as essential in nursing, enabling sensitive and appropriate responses to patients’ emotional needs. Therefore, training nursing students in empathy involves not only recognizing their own emotions but also recognizing and responding appropriately to the emotions of others [21]. This study represents a preliminary exploration of the TRI-COM model in the context of nursing education. While the framework provides a useful theoretical lens, further research is needed to validate its educational relevance and practical applicability.

### Scope

This study pursued two primary objectives:

(1) To introduce the TRI-COM model as a conceptual and educational framework to better operationalize the definition of emotions within nursing contexts. The model was not empirically tested or validated in this study but was used to frame the theoretical background and interpret the findings. The TRI-COM model, adapted from a multisystemic theory of emotion, was reformulated to structure emotional competencies in nursing education by integrating physiological activation, cognitive representation, and motivational regulation. This framework aims to bridge classical theories of emotion with the practical challenges of professional healthcare training, offering a structured perspective for future emotional education.
(2) To investigate the multidimensional structure of emotional competencies among nursing students, with particular attention to how certain psychological dynamics—such as the emergence of secondary alexithymic traits and possible learned helplessness—may hinder their development. This study explores how individual and contextual factors might influence emotional skills, especially under conditions of emotional overload or perceived lack of control, which are frequent in clinical training settings.

Based on the literature, it is hypothesized that repeated exposure to emotionally intense yet uncontrollable situations—such as persistent patient pain, death, ethical dilemmas, or lack of professional recognition—may foster a reduction in emotional self-efficacy, emotional detachment, and growing difficulties in recognizing, labeling, and regulating emotions. Over time, this may compromise the development of essential empathic and relational competencies.

In this context, the TRI-COM model was adopted solely as a conceptual framework to guide the interpretation of emotional competencies and to structure the discussion of empathy and alexithymia in nursing education. The present study does not aim to test or validate the TRI-COM model but rather uses it as a theoretical lens to contextualize findings and educational implications.

## 2. Materials and Methods

### 2.1. Procedure

This study was conducted on a sample of 233 undergraduate nursing students from various Italian universities that were evenly distributed across the three academic years. Data were collected between July 2021 and May 2022 using a convenience sampling strategy. Participation was voluntary and open to all eligible nursing students who had access to the survey link through university channels. Although the use of an online tool (Google Forms) presents some limitations in terms of identity verification, several measures were taken to ensure data reliability. The survey link was distributed exclusively through official academic channels, such as institutional mailing lists and course coordinators, targeting only enrolled nursing students. The platform prevented multiple submissions, and no incentives were offered, reducing the risk of random or duplicate responses.

### 2.2. Instruments

Data collection was carried out through an online questionnaire administered via Google Forms, consisting of three main sections:

- A sociodemographic section;
- The Toronto Alexithymia Scale—20 items (TAS-20) [22];
- The Jefferson Scale of Empathy—Health Profession Student (JSE-HPS) version [23].

These tools were selected based on their established psychometric properties and prior application in nursing education contexts.

Specifically, the TAS-20 is a widely validated self-report instrument composed of 20 items rated on a 5-point Likert scale (from 1 = strongly disagree to 5 = strongly agree). The TAS-20 measures three core dimensions of alexithymia: difficulty identifying feelings (DIF), difficulty describing feelings (DDF), and externally oriented thinking (EOT). A total score above 61 indicates clinically significant alexithymia. An example item from the TAS-20 is “I am often confused about what emotion I am feeling.” The TAS-20 demonstrates good internal consistency, with Cronbach’s α typically around 0.81 for the total scale (α = 0.67–0.84 across subscales), confirming its reliability across diverse populations [24]. The Italian language version of the TAS-20, validated by Bressi et al. [25], was used in this study.

The JSE-HPS version consists of 20 items rated on a 7-point Likert scale (from 1 = strongly disagree to 7 = strongly agree). The JSE-HPS evaluates empathy as a cognitive and behavioral construct, focusing on the ability to adopt the patient’s perspective and respond in a therapeutically meaningful way. The scale includes three subdimensions: Perspective Taking, Compassionate Care, and Walking in the Patient’s Shoes. Higher scores indicate greater levels of perceived empathy. An example item from the JSE-HPS is “I try to imagine myself in my patients’ shoes when providing care.” The JSE-HPS is considered one of the most reliable and widely used tools to assess empathy among health profession students [26,27]. This version is specifically designed for use with students in health professions, including nursing, and is distinct from the version intended for physicians. While the original validation study involved medical students and residents, the JSE-HPS has been widely used in research with nursing students and has demonstrated robust psychometric properties in these populations, with Cronbach’s α typically ranging from 0.76 to 0.89. The officially authorized and validated Italian version of the JSE-HPS, provided by the Jefferson Scale’s original developers, was used for this study.

### 2.3. Application of the Theoretical Framework

The TRI-COM model was not used as a direct measurement instrument, nor was it empirically tested in this study. Instead, it was adopted solely as a conceptual framework to guide the theoretical framing and interpretation of the findings. Specifically, the model helped in identifying alexithymia and empathy as key constructs of emotional competence relevant to nursing education. Its three components—physiological activation, cognitive representation, and motivational regulation—provided a conceptual lens through which to interpret the relationships between emotional processing, empathic functioning, and professional development. This study, therefore, does not validate the TRI-COM model, but rather uses it to contextualize the results within a comprehensive, multidimensional understanding of emotion in healthcare practice.

### 2.4. Ethical Considerations

This study was conducted in accordance with the ethical principles of the Declaration of Helsinki. Ethical approval for this study was obtained from the independent commission of the “Centro di Counseling psicologico”, University of Salerno (Italy) (number 01/2021, date 15 January 2021). Participation was voluntary, and informed consent was obtained from all participants before data collection. The online survey ensured anonymity, and no personal identifying information was collected. The participants were informed of their right to withdraw from this study at any time without any consequences.

### 2.5. Participants

The study sample consisted of 233 undergraduate nursing students enrolled in various Italian universities. The age range of participants was 19 to 54 years (mean age = 23.79, SD = 5.19). The distribution by university shows a clear predominance of students from the University of Salerno, who accounted for 58.4% of the total sample, followed by students classified under the aggregated category “Other Universities” (36.1%), which included several unspecified institutions. The remaining universities were represented in much smaller numbers (ranging from 0.4% to 1.7%), including the University of Campania “Luigi Vanvitelli”, University of Rome “La Sapienza”, University of Pisa, University of Bologna, University of Padua, University of Sassari, and University of Catanzaro.

Regarding the distribution by year of study, third-year students constituted nearly half of the sample (45.9%), followed by first-year (22.7%), second-year (20.6%), and out-of-course students (10.7%). This distribution suggests that the majority of participants were in an advanced stage of their academic training, likely having already engaged in meaningful clinical experiences, which is particularly relevant for investigating emotional and relational competencies.

Given that 82.8% of the participants identified as female, reflecting the gender distribution typical in the nursing profession, the sample is not gender-balanced. However, this composition is consistent with national nursing demographics and was expected. Nevertheless, it should be noted that the smaller proportion of male students may limit the generalizability of gender-related analyses, particularly in the detection of subtle gender differences in emotional competencies.

No data were collected regarding clinical comorbidities, psychological diagnoses, or ongoing treatments, as these were beyond the scope of this study.

No items had more than 5% missing data, ensuring robust data quality for all analyses.

### 2.6. Statistical Analysis

Data were analyzed using IBM SPSS (v.23). Descriptive statistics were computed to summarize demographic variables and the main psychometric scores (TAS-20 and JSE-HPS). Group comparisons were conducted using an independent sample t-test to examine gender differences in alexithymia and empathy scores. One-way ANOVAs were performed to assess differences in alexithymia and empathy across academic years. Pearson’s correlation coefficients were calculated to explore the relationships among age, academic year, alexithymia dimensions (DIF, DDF, EOT), and empathy components (Perspective Taking, Compassionate Care, Walking in the Patient’s Shoes). In cases of significant findings, post hoc comparisons were considered. Assumptions of homogeneity of variances were tested using Levene’s test. Additionally, chi-square tests were used for categorical associations when applicable. Significance was set at *p* < 0.05.

## 3. Results

The overall profile of the sample (see Table 1) reveals alexithymia scores approaching the clinical threshold, with a mean TAS-20 total score of 60.36 (SD = 11.22), just below the clinical cut-off of 61. Notably, a marked tendency toward externally oriented thinking (EOT, M = 26.48; SD = 3.16) was observed, compared with lower scores in difficulty describing feelings (DDF, M = 14.98; SD = 3.56) and difficulty identifying feelings (DIF, M = 18.88; SD = 5.52).

On the empathy side, scores from the Jefferson Scale of Empathy suggest a strong cognitive capacity for Perspective Taking (M = 60.95; SD = 7.42), but lower levels of compassionate emotional engagement (Compassionate Care, M = 18.92; SD = 6.40) and moderate ability to emotionally connect with the patient’s experience (Walking in the Patient’s Shoes, M = 7.18; SD = 2.65). The overall empathy score (JSE) was 87.05 (SD = 7.88), indicating a moderate level.

These findings, reported in the descriptive table for the general sample, suggest that the combination of alexithymic traits and only partially developed empathic competencies may represent a form of functional adaptation to the clinical environment. However, this profile raises important questions about the emotional training strategies that should be implemented in nursing degree programs to support the long-term development of relational and empathic skills.

The presentation of the results follows the order of this study’s stated objectives, beginning with general descriptive analyses, followed by gender differences, training level comparisons, and correlation patterns.

### 3.1. Gender Differences

An independent sample t-test was conducted to explore potential gender differences in alexithymia and its subcomponents (see Table 2). The results indicated no statistically significant differences between male and female students in the total TAS-20 score or its subscales (DDF, DIF, and EOT).

Specifically, female students reported a slightly higher mean TAS-20 total score (M = 60.74, SD = 11.38) compared with their male counterparts (M = 58.53, SD = 10.40), but the difference was not statistically significant, *t*(231) = 1.137, *p* = 0.257.

Regarding the subscales, females scored higher in difficulty describing feelings (DDF: M = 15.07, SD = 3.57) than males (M = 14.53, SD = 3.52), although, again, the difference was not significant, *t*(231) = 0.885, *p* = 0.377. Conversely, males showed slightly higher mean scores in difficulty identifying feelings (DIF: M = 19.00, SD = 4.47 vs. M = 18.86, SD = 5.72) and externally oriented thinking (EOT: M = 26.50, SD = 3.25 vs. M = 26.48, SD = 3.15), but these differences were negligible and not significant (DIF: *t*(231) = −0.146, *p* = 0.884; EOT: *t*(231) = −0.033, *p* = 0.974).

Although these results did not reach statistical significance, the observed trends are noteworthy. Male students appeared to report slightly greater difficulty in recognizing their emotions (DIF) and a stronger tendency toward externally focused, concrete thinking (EOT). These patterns may be interpreted in light of gendered socio-cultural models of emotional socialization. Traditional norms often discourage men from expressing or exploring emotions, favoring action-oriented and emotionally detached coping styles. This may account for the marginally higher EOT scores among male students, a subdimension of alexithymia that reflects limited emotional introspection.

Similarly, the slight increase in DIF scores among males may reflect reduced familiarity with emotional reflection or limited exposure to relational or educational settings that promote such skills. These findings underscore the importance of gender-sensitive emotional education in healthcare training programs.

Regarding empathic dimensions (see Table 3), female students demonstrated significantly higher scores in Perspective Taking (M = 61.54) compared with male students (M = 58.10), with a mean difference of +3.44 points (*p* = 0.007). This result corresponds to a moderate effect size (Cohen’s d = 0.47), suggesting a meaningful and practical difference in favor of female students.

Interestingly, the “Walking in the Patient’s Shoes” subscale, which assesses the imaginative ability to identify with the patient’s experience, was higher among male students (M = 7.93) than female students (M = 7.03), with a *p*-value at the threshold of statistical significance (*p* = 0.050). This result may reflect qualitative differences in how male and female students connect empathically with patients, although it does not support a clear-cut gender distinction.

No statistically significant differences were observed for the Compassionate Care subscale or the overall Jefferson Scale of Empathy (JSE) score. These findings suggest a general equivalence between genders in terms of affective empathy and overall empathic disposition within this sample.

### 3.2. Training Level

A one-way analysis of variance (ANOVA) was conducted to assess potential differences in empathy scores across academic years (see Table 4). The results did not reveal any statistically significant differences among students in the various years of the nursing program.

Specifically, the Perspective Taking subscale approached statistical significance, with F(3, 229) = 2.237, *p* = 0.085, suggesting a potential trend in perspective-taking ability across academic levels, although it did not meet the conventional threshold for significance (*p* < 0.05). The Compassionate Care (F(3, 229) = 1.020, *p* = 0.384) and Walking in the Patient’s Shoes (F(3, 229) = 2.116, *p* = 0.099) subscales also did not show significant differences across years, although the latter approached a level of potential interest for future analyses.

Finally, no significant differences were found in the total Jefferson Scale of Empathy (JSE) score across academic years (F(3, 229) = 0.839, *p* = 0.474), indicating a general consistency in perceived empathy regardless of the stage in the nursing curriculum.

The analysis of variance (ANOVA) conducted to examine potential differences in alexithymia (TAS-20) scores and its subscales across academic years did not reveal any statistically significant results (see Table 5). Specifically, for the difficulty describing feelings subscale (TAS20_DDF), the analysis yielded F(3, 229) = 0.100, *p* = 0.960, indicating no differences between groups. Similarly, for the externally oriented thinking subscale (TAS20_EOT), F(3, 229) = 0.209, *p* = 0.890, no meaningful variations across academic years were observed. The TAS-20 total score confirmed this trend, with F(3, 229) = 0.491, *p* = 0.689.

However, the difficulty identifying feelings subscale (TAS20_DIF) produced F(3, 229) = 2.604, *p* = 0.053, which, although not reaching the conventional threshold for statistical significance (*p* < 0.05), approaches it, suggesting a potential trend toward differentiation based on year of study. The near-significant trend in the DIF subscale (*p* = 0.053) was associated with a small effect size (η^2^ = 0.033), suggesting a modest proportion of variance explained by academic year.

### 3.3. Correlation Results

The correlation analysis revealed several significant relationships among the variables under investigation (see Table 6). First, age showed a significant negative correlation with all TAS-20 subscales: difficulty describing feelings (DDF) (*r* = −0.164, *p* = 0.012), difficulty identifying feelings (DIF) (*r* = −0.199, *p* = 0.002), and externally oriented thinking (EOT) (*r* = −0.143, *p* = 0.029).

The three TAS-20 subscales were strongly intercorrelated: DDF was positively associated with DIF (*r* = 0.502, *p* < 0.001) and EOT (*r* = 0.389, *p* < 0.001), while all three were significantly associated with the TAS-20 total score, with particularly high values for DDF (*r* = 0.829, *p* < 0.001) and DIF (*r* = 0.728, *p* < 0.001). EOT was also correlated with the total score, albeit more moderately (*r* = 0.613, *p* < 0.001).

On the empathy side, Perspective Taking was negatively associated with gender (*r* = −0.175, *p* = 0.007), indicating higher scores among female students. Furthermore, it showed a positive correlation with the total JSE score (*r* = 0.533, *p* < 0.001) and a negative correlation with the Compassionate Care subscale (*r* = −0.477, *p* < 0.001), a finding that may reflect a distinction between the cognitive and affective components of empathy.

The JSE total score was significantly correlated with all of its subscales, Compassionate Care (*r* = 0.431, *p* < 0.001), Perspective Taking (*r* = 0.533, *p* < 0.001), and Walking in the Patient’s Shoes (*r* = 0.439, *p* < 0.001), confirming the internal consistency of the scale.

Finally, significant correlations emerged between some alexithymia subscales and empathy dimensions: DDF and TAS-20 total were positively associated with Walking in the Patient’s Shoes (*r* = 0.179 and *r* = 0.177, respectively; both *p* < 0.01).

## 4. Discussion

### 4.1. Objective 1: To Propose a Conceptual Model to Operationalize the Definition of “Emotions” Within Nursing Contexts

The results of this study indicate that nursing students exhibit alexithymia scores close to the clinical threshold, particularly in the dimension of externally oriented thinking (EOT), alongside moderate difficulties in describing and identifying emotions. This profile suggests a general tendency toward emotional detachment and cognitive rationalization, which may reflect adaptive mechanisms developed within the context of emotionally intense clinical training.

One possible theoretical explanation for these findings lies in the TRI-COM model, which conceptualizes emotion as a multisystem process involving physiological activation, cognitive representation, and motivational regulation [28]. In this study, the TRI-COM model served only as a conceptual framework to interpret the data and guide the selection of constructs such as alexithymia and empathy. Within this perspective, alexithymia can be understood as a potential breakdown in the integration of these systems, particularly between bodily signals and their conscious representation.

However, it is important to note that the TRI-COM model was not empirically validated in this study. While it offers a promising structure for future educational interventions, its applicability in nursing education must be confirmed through dedicated empirical research.

Previous studies [29] have shown that nursing students often exhibit elevated alexithymic traits compared with general population norms [30], especially when educational settings lack structured emotional support. Nevertheless, alternative explanations should also be considered. For instance, the prevalence of alexithymic traits could be influenced by pre-existing personality factors [31] or by cultural norms that discourage emotional expression, particularly in professional healthcare settings [32]. Additionally, repeated exposure to emotionally demanding situations without adequate psychological support could contribute to emotional numbing or suppression [33].

Compared with classical models of emotional intelligence, such as Goleman’s or Mayer and Salovey’s frameworks, the TRI-COM model offers a systemic approach that better captures the complexity of emotional processing in high-stress, relationally intense contexts such as nursing. However, its integration into nursing curricula should currently be viewed as a future direction rather than a tested educational intervention.

### 4.2. Objective 2: To Investigate the Multidimensional Structure of Emotional Competencies Among Nursing Students

The data suggest a multidimensional profile of emotional competencies, characterized by relatively high scores in the cognitive dimension of empathy (Perspective Taking) but lower and more variable scores in the affective (Compassionate Care) and behavioral (Walking in the Patient’s Shoes) components. This pattern is consistent with prior research showing that empathy in healthcare students often develops more at the cognitive than at the affective or behavioral level [34].

Gender-based analyses revealed a statistically significant advantage for female students in Perspective Taking, a finding in line with the existing literature showing higher emotional sensitivity and perspective-taking abilities among women [35,36]. These differences may reflect broader socio-cultural patterns of emotional socialization, where women are generally encouraged to adopt more relationally oriented behaviors. However, no meaningful gender differences were found in other empathy components or alexithymia scores.

Interestingly, no significant variation was observed in empathy or alexithymia scores across academic years, which contrasts with some longitudinal studies reporting empathy decline over time [37]. This discrepancy may relate to program-specific factors, such as differences in the organization of clinical training, the availability of emotional supervision, or cultural variables that shape emotional learning.

Although one possible explanation for these results involves learned helplessness [7]—where repeated exposure to uncontrollable stressors can lead to emotional withdrawal—other interpretations are equally plausible. For example, emotional flattening might reflect protective desensitization to avoid overload or coping strategies developed in response to sustained exposure to suffering [29]. Likewise, the absence of significant differences across academic years could indicate gaps in formal emotional education, rather than reflecting a stable personal trait.

An intriguing pattern in the data suggests that greater difficulty in describing one’s own emotions (DDF) may paradoxically co-exist with a stronger tendency to engage in perspective-taking behaviors, such as “walking in the patient’s shoes.” This could reflect a compensatory mechanism or result from repeated exposure to emotionally intense clinical contexts, prompting empathic connection even in the absence of emotional clarity.

In this context, the TRI-COM model was used only as a conceptual framework to interpret these findings and organize the emotional dimensions investigated. While the model offers a useful lens to integrate neuropsychological, emotional, and behavioral aspects, it was not empirically validated in this study. Its potential utility in designing structured emotional education programs should therefore be regarded as a future research direction rather than a tested pedagogical outcome.

Finally, the negative correlations between age and all TAS-20 subscales (DDF, DIF, and EOT) suggest that emotional awareness and regulation may improve with age and cumulative experience. This interpretation aligns with recent findings showing that emotional intelligence tends to increase over time among nursing students [38]. Similarly, Sharafkhani et al. [39] observed that older nursing students scored lower in alexithymia, suggesting a greater ability to identify and express emotions. While these associations are promising, longitudinal studies are needed to confirm whether academic and clinical experiences directly contribute to the development of emotional competence.

### 4.3. Educational and Organizational Implications

The findings of this study underscore the need to integrate emotional training into nursing curricula in a structured, intentional, and assessable manner. The TRI-COM model provides a robust theoretical framework for such interventions, guiding educational design along three axes:

1. Theoretical modules covering neuropsychology of emotions, affect regulation, empathy, and stress management;
2. Experiential workshops using high-fidelity simulations, role-playing, and guided debriefing;
3. Reflective practice through emotional diaries, narrative supervision, and case discussions.

These strategies are aligned with best practices in nursing education and may be implemented without significantly increasing student workload. The three axes represent a strategic investment to prevent burnout, enhance professional satisfaction, and improve the quality of care [40]. Ultimately, emotional competencies should no longer be considered optional in healthcare training. Such competencies are essential for the development of ethically grounded, emotionally resilient, and clinically effective professionals.

From an organizational standpoint, applying the TRI-COM model could involve integrating emotional assessments into clinical evaluations and establishing ongoing reflective groups for students and tutors to support the continuous development of emotional competencies. Moreover, these tools should be viewed as foundational elements for developing ethically grounded, emotionally resilient, and clinically effective professionals—particularly in light of the challenges posed by increasingly complex care environments.

### 4.4. Limitations and Future Directions

This study has several limitations. First, the sample, although geographically varied, included a predominance of students from a single university and was mostly female. Therefore, a limitation of this study is the gender imbalance in the sample, with female students representing 82.8% of respondents, thus limiting the generalizability of gender-related findings. While this mirrors the actual gender distribution within the nursing profession in Italy, it may have constrained the statistical power to detect gender differences, particularly for male participants. Future research should aim to include more balanced samples or explore qualitative methodologies to better understand gender-specific emotional competencies in nursing education.

Second, the cross-sectional design of this study precludes any causal interpretation of the results. Third, reliance on self-report measures may introduce response bias and does not capture behavioral manifestations of emotional competence. Furthermore, no clinical data were collected regarding participants’ psychological history or treatment, which could be potential confounders.

Importantly, the TRI-COM model was used in this study only as a conceptual framework to guide the interpretation of results and not as a validated or empirically tested pedagogical tool. Future research should adopt longitudinal and mixed-method approaches, including observational and experimental designs, to further validate emotional competencies and, where appropriate, empirically test the applicability and effectiveness of the TRI-COM framework in nursing education.

Moreover, future research could benefit from integrating qualitative methodologies—such as narrative interviews, focus groups, or reflective diaries—to explore emotional experiences in a more contextualized and dynamic manner. This would enable a deeper understanding of how emotional challenges are processed by nursing students and professionals across different settings, specialties, and levels of experience.

## 5. Conclusions

This study provides preliminary insights into the emotional competencies of nursing students, revealing the presence of alexithymic traits—particularly externally oriented thinking—and a discrepancy between cognitive and affective dimensions of empathy. These patterns may reflect emotionally avoidant coping strategies that can emerge during clinical training in highly demanding settings.

The TRI-COM model was used exclusively as a conceptual framework to interpret these findings. It conceptualizes emotion as the interaction of physiological, cognitive, and motivational systems and offers a structured perspective to guide future research and educational interventions. However, the model itself was not empirically tested in this study and therefore requires further validation before its practical application in nursing curricula.

These findings underscore the relevance of structured emotional education in nursing programs. Developing emotional self-awareness, empathy, and regulation skills should be considered core educational objectives to foster resilience, relational competence, and quality of care.

Nevertheless, the conclusions must be interpreted with caution. The cross-sectional design, reliance on self-report measures, and the sample’s gender imbalance limit the generalizability of these results. Future studies should adopt longitudinal, mixed-method, and experimental approaches to confirm these findings and evaluate the effectiveness of integrating the TRI-COM framework into nursing education.

In summary, this study highlights the importance of structured emotional education and identifies the TRI-COM model as a promising but as yet unvalidated conceptual tool for fostering emotional competence in nursing students.

## Figures and Tables

**Table 1:** Descriptive statistics for alexithymia and empathy scores in the overall sample.

Variables	Mean	Standard Deviation	Theoretical Range/Cut-off
TAS_20_DDF	14.98	3.562	5–25
TAS_20_DIF	18.88	5.515	7–35
TAS_20_EOT	26.48	3.161	8–40
TAS_20_Score	60.36	11.224	20–100 (Cut-off for alexithymia ≥ 61)
Perspective_Taking (JSE)	60.95	7.422	10–70
Compassionate_Care (JSE)	18.92	6.400	5–35
Walking_in_the_Patient‘s_Shoes (JSE)	7.18	2.646	2–14
JSE_Score	87.05	7.876	20–140

Note: TAS-20 = Toronto Alexithymia Scale; DDF = difficulty describing feelings; DIF = difficulty identifying feelings; and EOT = externally oriented thinking. JSE = Jefferson Scale of Empathy; JSE subscales include Perspective Taking, Compassionate Care, and Walking in the Patient’s Shoes.

**Table 2:** Gender comparison on TAS-20 scores.

Alexithymia	M♀ ± SD	M♂ ± SD	*p* (Levene)	*t* (df)	*p* (Two-Tailed)	Mean Difference
TAS_20_DDF	15.07 ± 3.57	14.53 ± 3.52	0.859	0.885 (231)	0.377	+0.55 (♀ > ♂)
TAS_20_DIF	18.86 ± 5.72	19.00 ± 4.47	0.068	−0.146 (231)	0.884	−0.14 (♂ > ♀)
TAS_20_EOT	26.48 ± 3.15	26.50 ± 3.25	0.670	−0.033 (231)	0.974	≈0
TAS_20_Score	60.74 ± 11.38	58.53 ± 10.40	0.178	1.137 (231)	0.257	+2.21 (♀ > ♂)

Note: TAS-20 = Toronto Alexithymia Scale; DDF = difficulty describing feelings; DIF = difficulty identifying feelings; and EOT = externally oriented thinking.

**Table 3:** Comparison of the JSE subscale and total scores by gender.

Empathy	M♀ ± SD	M♂ ± SD	*p* (Levene)	*t*(df)	*p* (Two-Tailed)	Mean Difference
Perspective Taking	61.54 ± 7.14	58.10 ± 8.19	0.167	2.703 (231)	0.007	+3.44 (♀ > ♂)
Compassionate Care	18.62 ± 6.22	20.35 ± 7.14	0.985	−1.559 (231)	0.120	−1.73 (♂ > ♀)
Walking in Patient’s Shoes	7.03 ± 2.62	7.93 ± 2.66	0.435	−1.968 (231)	0.050	−0.90 (♂ > ♀)
JSE Total Score	87.19 ± 7.70	86.38 ± 8.77	0.416	0.592 (231)	0.554	+0.81 (♀ > ♂)

Note: JSE = Jefferson Scale of Empathy; JSE subscales include Perspective Taking, Compassionate Care, and Walking in the Patient’s Shoes.

**Table 4:** Variance analysis of Jefferson Scale of Empathy scores across academic years.

Scale	Sum of Squares	df	Mean Square	F	*p*-Value
Perspective Taking	363.92	3	121.31	2.237	0.085
	12,417.46	229	54.23		
	12,781.38	232			
Compassionate Care	125.36	3	41.79	1.020	0.384
	9378.09	229	40.95		
	9503.45	232			
Walking in the Patient’s Shoes	43.82	3	14.61	2.116	0.099
	1580.61	229	6.90		
	1624.43	232			
JSE Total Score	156.39	3	52.13	0.839	0.474
	14,236.09	229	62.17		
	14,392.48	232			

Note: One-way ANOVA results show no statistically significant differences in Jefferson Scale of Empathy scores across academic years. Perspective Taking and Walking in the Patient’s Shoes show near-significant trends (*p* = 0.085 and *p* = 0.099, respectively), suggesting potential developmental trajectories that may warrant further investigation in longitudinal studies.

**Table 5:** ANOVA of alexithymia scores across academic year.

Scale	Sum of Squares	df	Mean Square	F	*p*-Value
TAS_20_DDF	3.833	3	1.278	0.100	0.960
	2939.060	229	12.834		
	2942.893	232			
TAS_20_DIF	232.757	3	77.586	2.604	0.053
	6823.114	229	29.795		
	7055.871	232			
TAS_20_EOT	6.337	3	2.112	0.209	0.890
	2311.861	229	10.095		
	2318.197	232			
TAS_20_Score	186.890	3	62.297	0.491	0.689
	29,040.827	229	126.816		
	29,227.717	232			

Note: One-way ANOVA results indicate no statistically significant differences in alexithymia scores across academic years. However, the DIF subscale (difficulty identifying feelings) approaches significance (*p* = 0.053), suggesting a possible trend in the development of emotional identification skills over the academic trajectory.

**Table 6:** Correlation matrix of alexithymia, empathy, and related variables.

Variables	Age	TAS_20_ DDF	TAS_20_ DIF	TAS_20_ EOT	TAS_20_ Score	Walking in the Patient’s Shoes (JSE)	Perspective Taking (JSE)	Compassionate Care (JSE)	JSE Total Score	Gender
Age	—									
TAS_20_DDF	−0.164 *	—								
TAS_20_DIF	−0.199 **	0.502 ***	—							
TAS_20_EOT	−0.143 *	0.389 ***	0.246 ***	—						
TAS_20_Score	−0.232 ***	0.829 ***	0.728 ***	0.613 ***	—					
Walking in the Patient’s Shoes (JSE)	−0.096	0.179 **	0.151 *	0.092	0.177 **	—				
Perspective Taking (JSE)	−0.066	0.059	0.041	−0.076	−0.008	0.184 **	—			
Compassionate Care (JSE)	−0.069	0.055	−0.008	0.017	0.035	0.201 **	−0.477 ***	—		
JSE Total Score	−0.043	0.088	0.045	−0.040	0.049	0.439 ***	0.533 ***	0.431 ***	—	
Gender	0.017	−0.107	−0.019	0.005	−0.059	−0.014	−0.175 **	0.010	−0.104	—

Note: Values are Pearson’s correlation coefficients (two-tailed). DDF = difficulty describing feelings; DIF = difficulty identifying feelings; and EOT = externally oriented thinking; JSE = Jefferson Scale of Empathy. * *p* < 0.05, ** *p* < 0.01, and *** *p* < 0.001.

## Data Availability

The data presented in this study are available from the corresponding author upon request due to privacy reasons.
